# Classification of unlabeled online media

**DOI:** 10.1038/s41598-021-85608-5

**Published:** 2021-03-25

**Authors:** Sakthi Kumar Arul Prakash, Conrad Tucker

**Affiliations:** 1 Department of Mechanical Engineering, Carnegie Mellon University, 5000 Forbes Avenue, Pittsburgh, PA 15213-3890 USA; 2 Department of Machine Learning, Carnegie Mellon University, 5000 Forbes Avenue, Pittsburgh, PA 15213-3890 USA; 3 The Robotics Institute, Carnegie Mellon University, 5000 Forbes Avenue, Pittsburgh, PA 15213-3890 USA; 4 Department of Biomedical Engineering, Carnegie Mellon University, 5000 Forbes Avenue, Pittsburgh, PA 15213-3890 USA; 5 CyLab Security and Privacy Institute, Carnegie Mellon University, 5000 Forbes Avenue, Pittsburgh, PA 15213-3890 USA

**Keywords:** Engineering, Mathematics and computing, Computer science

## Abstract

This work investigates the ability to classify misinformation in online social media networks in a manner that avoids the need for ground truth labels. Rather than approach the classification problem as a task for humans or machine learning algorithms, this work leverages user–user and user–media (i.e.,media likes) interactions to infer the type of information (fake vs. authentic) being spread, without needing to know the actual details of the information itself. To study the inception and evolution of user–user and user–media interactions over time, we create an experimental platform that mimics the functionality of real-world social media networks. We develop a graphical model that considers the evolution of this network topology to model the uncertainty (entropy) propagation when fake and authentic media disseminates across the network. The creation of a real-world social media network enables a wide range of hypotheses to be tested pertaining to users, their interactions with other users, and with media content. The discovery that the entropy of user–user and user–media interactions approximate fake and authentic media likes, enables us to classify fake media in an unsupervised learning manner.

## Introduction

The idea that people may not be suitable to assess the authenticity of information without the aid of additional tools is widely explored by the forensics community^1,2^. Information sharing in social media is highly dependent on data modality, the behavior of fellow networkers, the habit of using online services, and the widespread presence of opinion leaders in posts and comments^3,4^. In some cases, information is doctored using *deep fakes*^5^, or manipulated by spreading misinformation relating to health outbreaks such as COVID-19^6,7^. Beyond affecting the conduit of social media networks, much of algorithmic fake news detection^2,8^ or recommender systems^9^ rely on data type identification and data labeling. Such information is typically manually compiled by content moderators in social media companies who may undergo post-traumatic stress disorder as a result of viewing disturbing media^10,11^. Data-driven supervised methods benefit from superior classification accuracy due to the availability of labels but may fail to generalize to unseen data and often rely on manual human labeling. Alternatively, unsupervised methods do not rely on labels to classify data and address the trade-off in accuracy with the help of additional features, feature representation techniques, or complex models. Thus, there is a necessity to shift towards unsupervised approaches for content discovery or fake media discovery^12–14^.

In this study, we consider (1) work that seeks to understand why misinformation spreads and (2) work that seeks to prevent the spread of misinformation through classification. Current literature has improved our understanding of truth and falsity diffusion through quantitative analyses on the spread of rumors such as the discovery of the Higgs boson^15^, and the spread of anti-vaccine campaigns that led to decreases in vaccination rates against measles^16,17^. Other studies have investigated rumor diffusion modeling^18^, credibility evaluation^19,20^ and intervention strategies to curtail the spread of rumors^21^. Similar analyses have been conducted in social media networks such as Facebook, where Del Vicario et al.^22^ analyze the diffusion of science and conspiracy-theory stories, and in Twitter where Bovet and Kakse^23^ study the influence of fake news during the 2016 presidential election, and Vosoughi et al.^24^ study the characteristics of fact-checked rumors.

The spread of information, authentic or fake, is however significantly impacted by the credibility of the users in the network and their connections^25^. In a social media network such as Twitter, users form opinions and beliefs^26^ based on outside influences^23,27^, and use these opinions to test if information adheres to or opposes their beliefs^28^. Though the most direct measure of user credibility involves asking the other users^29^, such a measure is often subjective. Additionally, a subjective measure does not apply to sources or users previously unknown to a user^30^. However, such occasions are likely to occur during times of disasters such as COVID-19^7^, or during measles outbreaks^16,17^ where authentic information was provided by those physically attending the event^30^. Hence, we are interested in understanding how fake information spreads in a controlled social media network where users respond to unknown users with the help of an objective credibility measure.

Node-edge homogeneity and the homogeneous sharing paths created by social media users during the evolution of a social media network, play a critical role in the spread of fake information^22,31,32^. It has been shown that users’ opinions on media and their engagement behaviors on social media networks can be utilized to distinguish between authentic and fake information^8,33,34^. However, such work^8,33,34^ typically makes use of supervised learning techniques to detect fake information, requiring a labeled dataset. Though these supervised methods have shown promising results, they suffer from a critical limitation of requiring a pre-annotated dataset to train a classification model. Further, Chu et al.^35^ and Wang^33^ have released Facebook and Twitter datasets that include user activity and interactions pertaining to a specific time period, thereby not capturing the organic growth of user–user connections from the time users join the network. In order to address the limitations of requiring human-annotated datasets to train supervised models, crowd-sourcing approaches make use of platforms such as Amazon Mechanical Turk to recruit workers as users or as data labelers to take part in social media experiments. Crowd-sourcing techniques typically leverage cost-effective workers to obtain annotations, hence alleviating the burden of expert checking^36^. Long et al.^37^ and Rodrigues et al.^38^ propose a Bayesian probabilistic approach coupled with active learning, wherein they use crowd-sourced data to estimate both the data authenticity as well as the credibility of each individual data labeler/user. Recent studies^39,40^ have proposed replacing the Expectation-Maximization (EM) algorithm which is typically used for parameter estimation and inference with a deep generative model such as a variational auto-encoder (VAE). By replacing the EM algorithm with neural networks, the additional computational overhead can be avoided, which allows the models to generalize beyond classification settings^40^. Rodrigues et al.^40^ introduce an additional layer (crowd layer) in their CNN such that the typical softmax output layer is transformed into a bottleneck layer while allowing the crowd layer to handle data labeler/user reliability and labeling noise. While deep neural networks have been shown to improve the classification/labeling accuracy of unsupervised probabilistic models that use EM, the notion of explainability is replaced by a black-box approach. A study by Yang et al.^14^ proposes an unsupervised approach for detecting misinformation in online social media networks such as Twitter and Facebook using yet another probabilistic graphical approach. In their approach, Yang et al. consider the credibility of users as a latent random variable in addition to user opinions and use an efficient Gibbs sampling approach to estimate news authenticity. While supervised approaches such as^8,33,34^ have leveraged user–user interactions, these unsupervised probabilistic approaches^14,38–40^ explicitly assume that each user’s opinion is independent of other users. Hence, they do not consider user–user interactions, which can be an influential source of recommendation in Twitter and Facebook^41–43^, especially if users know one another. Further, prior work^14,38–40^ considers news features^39^ as a latent random variable in addition to considering the authenticity of news/data and the credibility of the data labelers/users^37,40^ as latent random variables.

When presented with uncertainty, human behavior in social media networks tends to react differently, employing unconventional social heuristics^44^. Hence, regardless of whether users know one another, there exists uncertainty in what a user likes and who they follow or befriend. The use of information-theoretic measures, such as entropy, avoids making assumptions about human behavior, thus allowing statistical characterization of uncertainty^45^ in social media networks. Shannon’s entropy has been used as a statistical feature in detecting rumors in social media^46^, abnormal activities or anomalies in IoT devices and sensor networks^47–49^. In addition to detecting anomalies, entropy has also been used in determining the credibility of machines in a machine-machine communications network in order to distinguish malicious nodes from honest nodes by iteratively updating the credibility value of each node for every message passed between the nodes^50^. Similarly, entropy has also been used to estimate the veracity of topics from tweets, such a measure has been reported to categorize tweets in the veracity spectrum in a consistent manner^51^.

Since it is evident that users in social media networks adhere to a notion of credibility before following a user and liking media, we derive a relationship between an objective credibility measure, user opinions (media likes), and the probability of following or establishing a connection with a user. We show that such a relationship helps understand the connection between user opinions and the credibility of users, and how they affect the probability of users making new connections. To that end, unlike prior approaches^14,38–40^, in our work, we do not consider the authenticity/label of the data as a latent variable, but instead only consider user opinions as a latent variable. This avoids the assumption that users like and share media on the basis of truth when users could intentionally like or share media on the basis of satire^52^. Additionally, the derived proportionality between user credibility and user opinion allows us to use one in place of another, thereby decreasing the number of latent variables considered. Then, we compute the entropy of user–user connections given user opinions in order to map user interactions to a quantifiable value that expresses the uncertainty contributed to the network by each user. Further, by exploiting the principle of entropy, we select users based on a threshold entropy to take part in a majority voting in order to distinguish fake information from authentic information. This allows utilizing users and their interactions as features to detect misinformation while decoupling the estimation of credibility or opinion and classification of data.

The paper is presented as follows. First, we annotate the controlled experiment conducted to collect real-time social network data. Then, we derive a relationship between user credibility, media likes, and the probability of establishing a connection with a user, followed by a comprehensive analysis that shows how entropy explains the spread of fake information. Finally, we propose an unsupervised model that uses entropy to select users to classify information as fake or authentic.

## Methods

### Data collection

**Figure 1:**
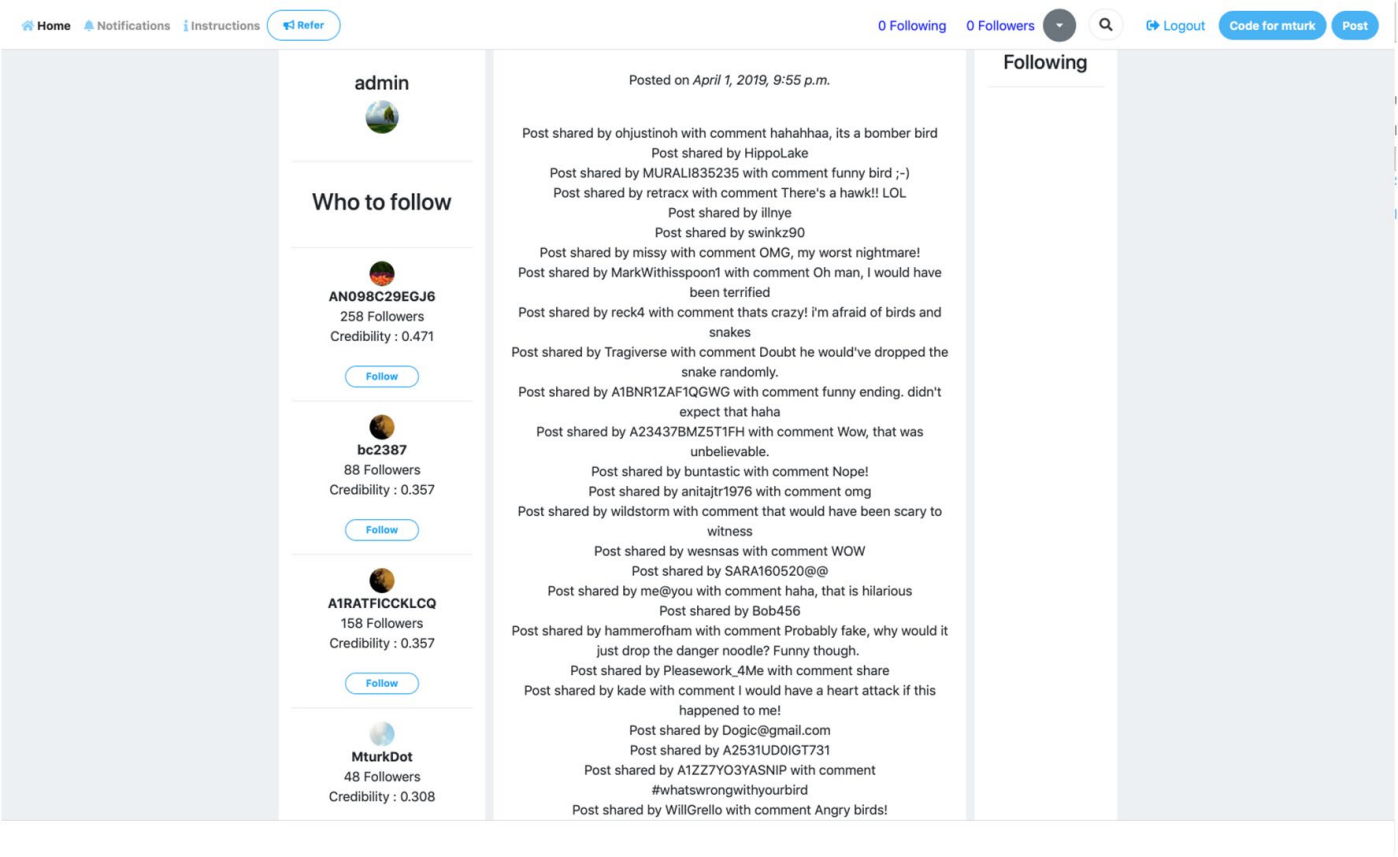
The user interface of the designed experimental platform that replicates the functionality of Twitter social media network.

We conduct a real-life social experiment, wherein participants are recruited from the crowd-sourcing platform *Amazon MTurk* (AMT) to participate in the study. The study was conducted with the approval of and in accordance with the relevant guidelines and regulations of The Pennsylvania State University’s *Institutional Review Board* (IRB). Prior to the conduct of the study, informed consent was obtained from all participants for participation in the study. Only human subjects of age 18 or above were included in the study. We created a mock online social media platform that replicates the functionality of Twitter as shown in Fig. 1 and follows their directional model of interactions. We restrict users from posting their own media and from unfollowing a followed user to conduct a controlled real-time, real-world social experiment. This web application is released for public use to replicate or conduct similar online social experiments. The focus of the data collection is to organically acquire the interactions between unknown users rather than from known users within the network in order to gain a fundamental understanding of how fake information can be classified using random user opinions. We populate the social network with *authentic* and *fake* videos from the *FaceForensics* data set^53^ and verified fake YouTube videos. The users were monetarily incentivized to spend at least 45 min per Human Intelligence Task (HIT). To ensure that the human subjects are reliable in staying throughout the study and with a provision for new users to join, we only recruited subjects who had a HIT’ (Human Intelligence Task) approval rate of greater than 90% in all requesters’ HITs. A user was allowed to participate in any number of HITs over the period of study, ensuring that the same user population could log into the network and continue accruing credibility and followers. We chose to use 40 random videos, with 20 being authentic and the other 20 fake. The total number of enrolled users in the study was 620, which falls within the range of participants recruited for other social science experiments that utilize AMT^54,55^.

**Figure 2:**
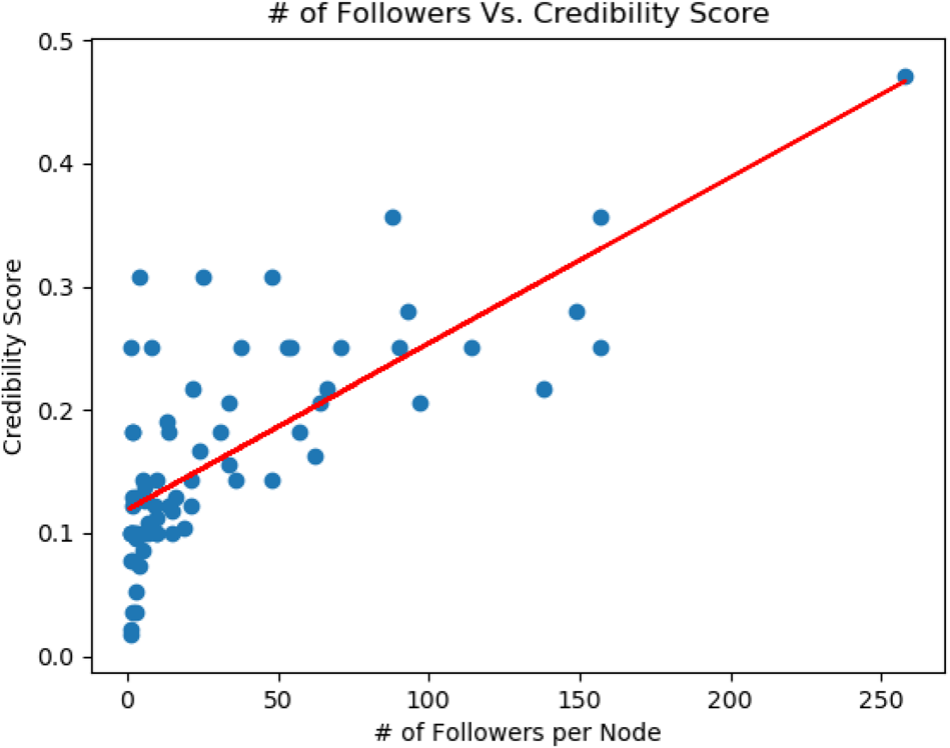
Fitted regression curve to follower counts against credibility score.

**Figure 3:**
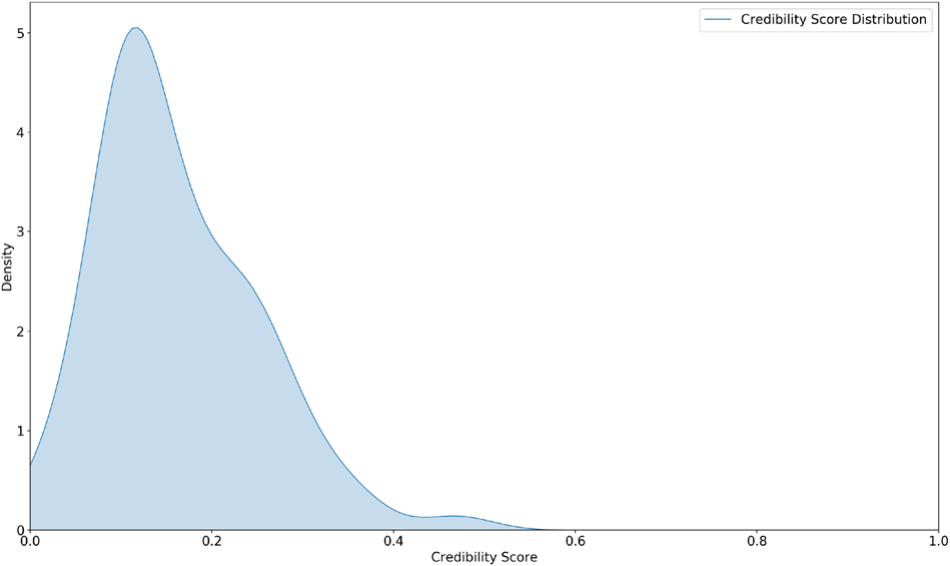
Credibility score distribution.

To simulate a real-world scenario in Twitter where users follow users or are followed by users thereby forming explicit connections^56^, we introduce the notion of user credibility. According to Resnick et al.^57^, reputation mechanisms provide an incentive for honest behavior and help users make decisions involving trust. In this study, we develop an approach to compute user credibility using trust modeling. Specifically, we use the Beta Reputation System (BRS)^58^ used in e-commerce to calculate the credibility of users. We select this method as it is grounded in probability theory and uses expectations on probability distributions to derive the predicted benefit of trust to help users form explicit connections in a participatory media setting. The credibility of user *i* is $C_{i}$ and we assume the prior probability of $C_{i}^t$ at time *t* to be generated from a Beta distribution with parameters $a^{t}$ (prior authentic media category counts) and $b^{t}$ (prior fake media category counts) such that $C_{i}^t$ $\sim ~Beta$($a^{t}$, $b^{t}$). We use the uniform distribution as prior to the Beta distribution such that every new user is assigned a credibility score of 0.1. As the user continues to form new links within the network, and like media, the credibility score gets updated. We use a Bayesian update rule to update the credibility score of the user at each timestamp. We state the credibility update rule for user *i* such that the parameter $a^t$ is updated as $a^{t+1}_i \leftarrow a^{t}_i + a^{\delta t}_i$ and $b^t$ is updated as $b^{t+1}_i \leftarrow b^{t}_i + b^{\delta t}_i$ such that,

1 $$\begin{aligned} {C_{i}^{(t+1)}} = \frac{a^{t}_i + a^{\delta t}_i}{a^{t}_i + a^{\delta t}_i + b^{t}_i + b^{\delta t}_i} \end{aligned}$$

where $\delta t$ is 20 min. To understand the relationship between credibility and follower count, we fit a regression model between follower counts and user credibility as shown in Fig. 2 and find the co-efficient of determination ($R^2$) as 0.56. This shows that there is a positive correlation between follower count and credibility score. Additionally, we also compute and illustrate the final credibility score distribution of the social media network at the last timestamp as shown in Fig. 3. We find that the distribution closely fits the Beta distribution given the uniform prior with ${\mathbb {E}}[C] = 0.1$. We also provide a monetary incentive to the top three users who earn the highest credibility score and get the highest user following by the end of the 2-day experiment.

### Preliminaries and definitions

The social network under study is a directed network having links specifying the followers (in-degree) and the following (out-degree). A social network ${\mathscr {G}}(t):=\{{\mathscr {V}},{\mathscr {E}}\}$ is an order 2 Tensor such that ${\mathscr {G}}^{t} \in {\mathbb {R}}^{N \times N}$ where *N* represents the number of users or ${\mathscr {V}}$ such that ${\mathscr {V}} \in {\mathbb {R}}^N$, ${\mathscr {E}} := \{(i,j) | i,j \in {\mathscr {V}}, i \ne j, {\mathscr {E}} \subseteq {\mathscr {V}} \times {\mathscr {V}}\}$ is the set of all pairs of distinct nodes, called edges and $t \in {\mathscr {T}}$ denotes timestamp index when all network information is saved in the database. Let the degree matrix **D** of ${\mathscr {G}} = diag(d(1),\ldots ,d(N))$ and **A** denote the adjacency matrix such that $A_{ij} = 1$ if and only if *i* follows *j* in case of out-degree network (${\mathscr {G}}_{out}$) or *j* is followed by *i* in case of in-degree network (${\mathscr {G}}_{in}$). The media liked by node *i* is denoted as $\mathbf{Z }_{i}$ which is a one-hot vector, where $\mathbf{Z }_{i}=[Z_1,Z_2,\dots , Z_L]$ and $Z_l$ denotes the user *i* liking category *l* such that $Z_{l} = 0$ indicates no like and $Z_{l} = 1$ indicates media like.

### Relating entropy to user credibility

We assume that users recruited in our social network are not known to each other and by showing every user the credibility of all users, we assume that users tend to follow or be followed by other users on the basis of credibility. We observe a positive linear correlation between the number of links connected to a user (in-degree) and credibility as shown in Fig. 2, consistent with findings from other works^30^. However, in reality, such a credibility measure is often network-dependent, or may not be readily available. Hence, we use links and media likes to approximate user credibility. We define a similarity metric, *Media Node-Edge Homogeneity*, which computes similarity as a function of common media liked by any two users, which have an edge connection at time *t*. We denote the media node-edge homogeneity (MNEH) metric as $M_{ij}^{t}$, where,

2 $$\begin{aligned} {M_{ij}^{t}} = \frac{|\mathbf{Z }_{i}^{t} \cap \mathbf{Z }_{j}^{t}| }{|\mathbf{Z }_{i}^{t} \cup \mathbf{Z }_{j}^{t}|} \end{aligned}$$

In Eq. (2), $M_{ij}^{t}$ between any two users *i* and *j* at time (*t*), is bounded between the interval [0, 1], where, $0 \le M_{ij} \le 1$ expresses the magnitude of similarity in media *likes* between any two users *i* and *j*. The node-edge homogeneity interval [0, 1] is discretized with a resolution of 0.01. The MNEH matrix ($\mathbf{M }^t$) is a sum of matrices as shown in Eq. (3) with varying $\gamma$, such that ${\tilde{\gamma }} = \gamma /100$, $M_{ij,\gamma }^{t} = 1$ if and only if the similarity in media liked by users *i* and *j* is $\gamma$%.

3 $$\begin{aligned} {\mathbf{M }^{t} =\sum _{\gamma =1}^{100}{\tilde{\gamma }}\mathbf{M }_{{\tilde{\gamma }}}^{t}} \end{aligned}$$

Given $\mathbf{A }^t$ and $\mathbf{M }^{t}$ from the in-degree and out-degree networks, we assess the Directed Acyclic Graph (DAG) connecting *i*, *j* and $\mathbf{M }^{t}$. We assume liking media and forming links with other users to be conditionally independent events since $j=1$ is observed thereby breaking the dependence between the events. Hence, we compute the probability of a link between user *i* and *j* for each instant in time *t* as shown in Eq. (4).

4 $$\begin{aligned} \begin{aligned} p(j=1|{\mathbf{M }}_{{\tilde{\gamma }}}^{t},i)&= p(j=1|i).p(j=1|{\mathbf{M }}_{{\tilde{\gamma }}}^{t}) \\&= {(\mathbf{A }\mathbf{D }^{-1})}_{ij}^{t}.\frac{p(j=1,{\mathbf{M }}_{{\tilde{\gamma }}}^{t})}{p({\mathbf{M }}_{{\tilde{\gamma }}}^{t})}\\&= {(\mathbf{A }\mathbf{D }^{-1})}_{ij}^{t}.{(\mathbf{M }_{{\tilde{\gamma }}}\mathbf{D }^{-1}_{\mathbf{M }_{{\tilde{\gamma }}}})}_{ij}^{t} \\&= {((\mathbf{A }\circ \mathbf{M }_{{\tilde{\gamma }}})\mathbf{D }^{-1}_{\mathbf{A }\mathbf{M }_{{\tilde{\gamma }}}})_{ij}^{t}} \\&= P_{ij}^{t}.P_{ij,{\tilde{\gamma }}}^{t}\\ \end{aligned} \end{aligned}$$

where $p(j=1|i) = P_{ij}^{t}$ is a conditional probability which represents the probability of a link formed with user *j* given user *i* at time *t* such that $P_{ij}^{t}$ is an element of the transition matrix ($\mathbf{P }^{t}$) which is algebraically computed as $(\mathbf{A }\mathbf{D }^{-1})^{t}$, and $\frac{p(j=1,{\mathbf{M }}_{{\tilde{\gamma }}}^{t})}{p({\mathbf{M }}_{{\tilde{\gamma }}}^{t})} = P_{ij,{\tilde{\gamma }}}^{t}$ is computed as follows,

5 $$\begin{aligned} \begin{aligned} \frac{p(j=1,{\mathbf{M }_{{\tilde{\gamma }}}^{t}})}{p({\mathbf{M }_{{\tilde{\gamma }}}^{t}})}&= \frac{1~\{{M_{ij,{{\tilde{\gamma }}}}^{t}}=1\}}{\sum _{j\in N}~1~\{{M}_{ij,{\tilde{\gamma }}}^{t}=1\}} \\&= {(\mathbf{M }_{{\tilde{\gamma }}}\mathbf{D }^{-1}_{\mathbf{M }_{{\tilde{\gamma }}}})}_{ij}^{t} \\&= P_{ij,{\tilde{\gamma }}}^{t} \\ \end{aligned} \end{aligned}$$

where ${\mathbf{D }}_{{\mathbf{A }\mathbf{M }_{{\tilde{\gamma }}}}}$ is a diagonal matrix such that ${D}_{{\mathbf{A }\mathbf{M }_{{\tilde{\gamma }}}},ii} = \sum _{j \in N}(A\circ ~M)_{ij,{{\tilde{\gamma }}}}$, ${\mathbf{D }}_{{\mathbf{M }_{{\tilde{\gamma }}}}}$ is a diagonal matrix such that ${D}_{{\mathbf{M }_{{\tilde{\gamma }}}},ii} = \sum _{j \in N}~M_{ij,{{\tilde{\gamma }}}}$ and ($\circ$) denotes Hadamard product operation between matrices. For each media category $l \in L$, $Z_{jl}^t$ is generated from a Bernoulli distribution with parameter $C_{j}^t$ such that $Z_{jl}^t$ $\sim ~Bernoulli$($C_{j}^t$) and $C_{j}^t$ is the probability of user *j* liking *l*. Hence, we assume the prior probability of $C_{j}^t$ to also be the assumed credibility of a user *j* and sampled from a Beta distribution with parameters $a^{t}$ (prior authentic media category counts) and $b^{t}$ (prior fake media category counts) such that $C_{j}^t$ $\sim ~Beta$($a^{t}$, $b^{t}$). In a similar manner as Eq. (4), we compute the probability of a link between user *i* and *j* given the media matrix ($\mathbf{Z }_{j}^{t}$) for each instant in time *t* as shown in Eq. (6)

6 $$\begin{aligned} \begin{aligned} p(j=1|{{\mathbf{Z }}_j^t,i})&= p(j=1|i).p(j=1|{{\mathbf{Z }}_j^t}) \\&= P_{ij}^t.\int {p(j=1,C_{j}^t|\mathbf{Z }_{j}^t)dC_{j}^t} \\&= P_{ij}^t.\int {p(j=1|C_{j}^t).p(C_{j}^t|\mathbf{Z }_{j}^t)dC_{j}^t} \\&= \frac{P_{ij}^t}{\mathbf{B }(a^{t},b^{t})}\int {C_{j}^{Z_{jl}^t+{a^{t}}-1}.(1-{C_{j}^t})^{(1-{Z_{jl}^{t}})+{b^{t}}-1}dC_{j}^t} \\&= P_{ij}^t.\frac{\mathbf{B }({Z_{jl}^t+{a^{t}}}, {Z_{jl}^t+{b^{t}}})}{\mathbf{B }(a^{t}, b^{t})} \\&= P_{ij}^t.\left( \frac{a^t}{a^t + b^t}\right) \\&= {P_{ij}^{t}}.{C_{j}^{t}} \\ \end{aligned} \end{aligned}$$

where $\mathbf{B }{(.)}$ is the Beta function and normalizes the expression in Eq. (6). Let $p(j=1|{{\mathbf{Z }}_j^t})$ be denoted by $\Omega (Z)$ and $p(j=1|{{\mathbf{M }}_j^t})$ be denoted by ${\mathscr {F}}(\Omega (Z))$ since ${{\mathbf{M }}_j^t}$ is a function of ${{\mathbf{Z }}_j^t}$. Using the results from Eqs. (4) and (6), we derive the proportionality between $C_j^t$ and $P_{ij,{\tilde{\gamma }}}^t$ given ${\tilde{\gamma }}$ for every timestamp as shown by Eq. (7).

7 $$\begin{aligned} \begin{aligned} \frac{p(j=1|i).p(j=1|{{\mathbf{Z }}_j^t})}{C_j^t}&= \frac{p(j=1|i).p(j=1|{\mathbf{M }}_{{\tilde{\gamma }}}^{t})}{P_{ij,{\tilde{\gamma }}}^t}\\ \frac{p(j=1|i).\Omega (Z)}{C_j^t}&= \frac{p(j=1|i).{\mathscr {F}}(\Omega (Z))}{P_{ij,{\tilde{\gamma }}}^t}\\ P_{ij,{\tilde{\gamma }}}^t&\propto C_j^t \\ \end{aligned} \end{aligned}$$

Hence, from Eq. (4), it is evident that we are indeed computing a weighted transition probability with the weight being proportional to the user’s credibility. Using these results, we now compute the Shannon’s Information Entropy (joint entropy) generated by all users (*i*, *j*, *k*, ..., *n*) as the sum of entropy generated by each user at time *t*.

8 $$\begin{aligned} {H_{i,j,k,\dots ,n,{\tilde{\gamma }}}^t = -\sum _{i}^{N}\sum _{j}^{N}(P_{ij}^t.P_{ij,{\tilde{\gamma }}}^t)\log _{2}(P_{ij}^t.P_{ij,{\tilde{\gamma }}}^t)} \end{aligned}$$

## Results and discussion

**Figure 4:**
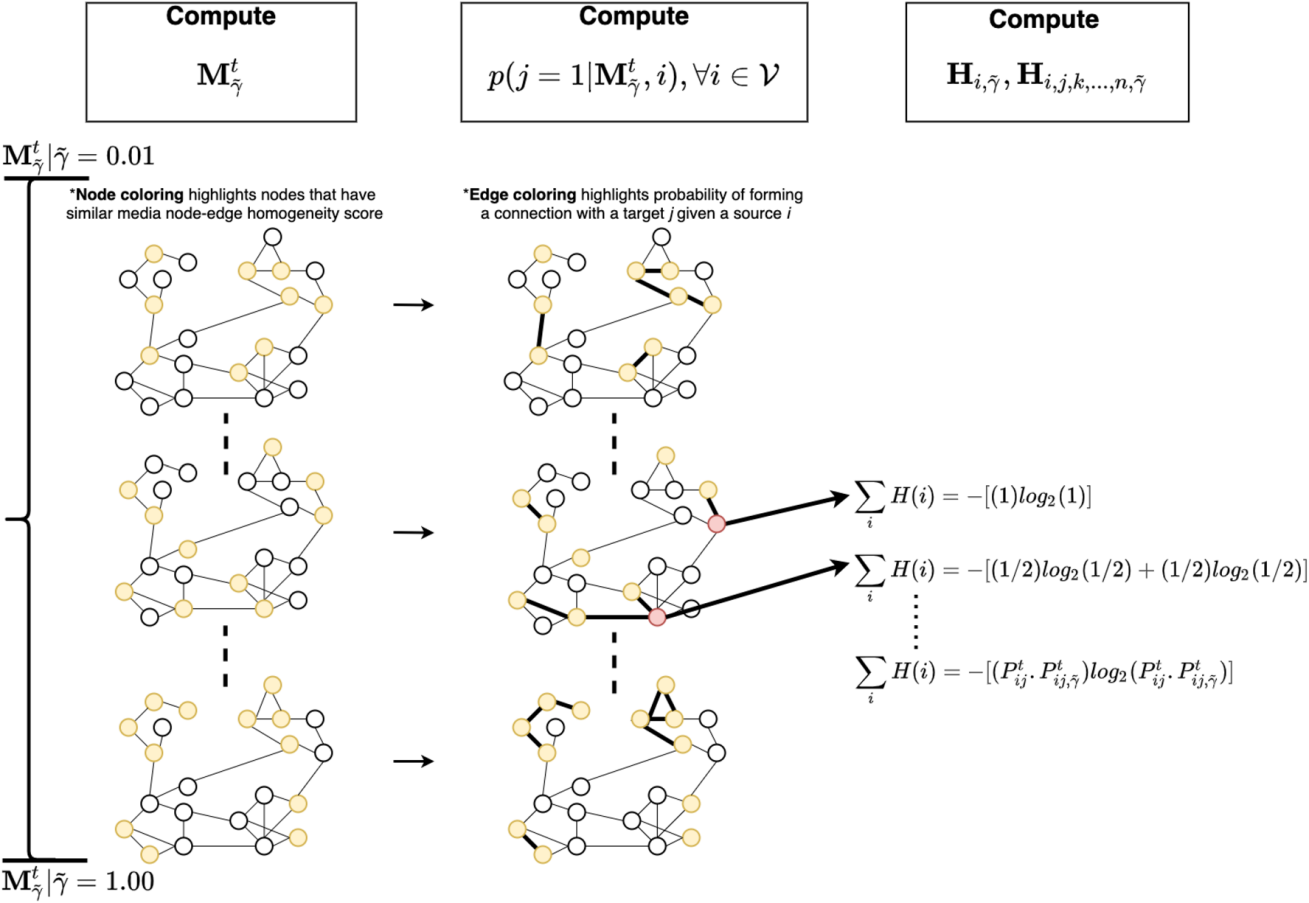
Social media network at time *t*. The figure presents a visual overview of the steps involved in computing the MNEH matrix ($\mathbf{M }^{t}_{{\tilde{\gamma }}}$) when ${\tilde{\gamma }}$ varies between 0.01 and 1.00, then computing the probability of forming a connection with user *j* given user *i*, and the entropy contribution of target nodes (colored red).

### Objective

The objective of this study is to use the entropy response of media node-edge homogeneity over time to perform unsupervised classification of fake media. We present a visual overview of our entropy computation approach as shown in Fig. 4, along with an algorithm box (Algorithm 1). We have organized this section to discuss hypotheses, hypotheses testing, and how the tests inform the selection of users that separate fake and authentic media in a majority voting classifier to classify fake media. Hereafter, the notations $H_0, H_{0*}$ and $H_a, H_{a*}$ refer to null-hypothesis and alternate hypothesis respectively.

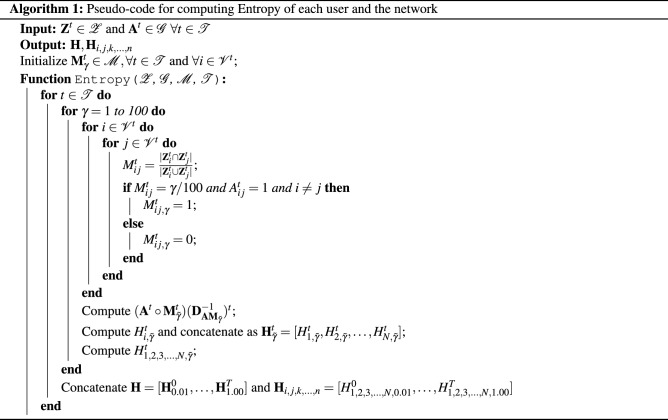



### Entropy of media node-edge homogeneity

**Figure 5:**
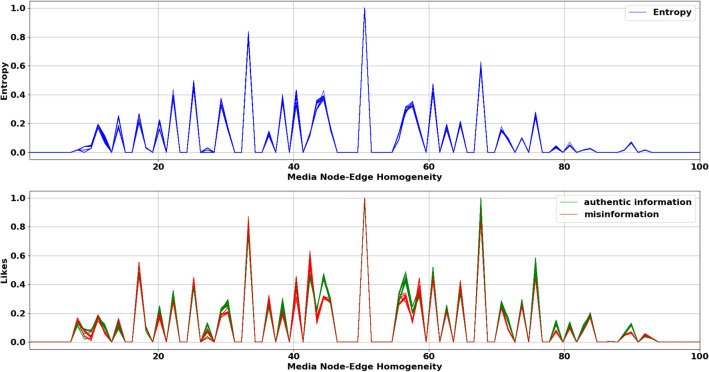
Normalized entropy response and normalized likes distribution against media node-edge homogeneity in the in-degree network.

**Figure 6:**
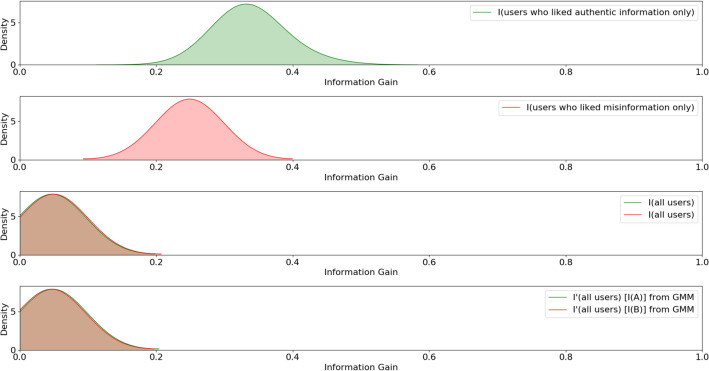
(**a**) Gaussian KDE of in-degree users who have only liked authentic media, (**b**) Gaussian KDE of in-degree users who have only liked fake media, (**c**) Gaussian KDE of all in-degree users who have liked authentic and fake media, (**d**) GMM approximation of information gain from authentic and fake media.

We compute the joint information entropy of the matrix for each value of ${\tilde{\gamma }}$ and for each *t* using Eq. (7). The social media network is directional as it has an in-degree or followee network and an out-degree or follower network. Hence, we hypothesize that the entropy generated by a random variable ($\mathbf{X }$) representing social network users in the in-degree and the out-degree network is sampled from the same underlying node-edge distribution, formally stated as follows:

- $$H_0$$: $$H(\mathbf{X })_{in} = H(\mathbf{X })_{out}$$.
- $$H_a$$: $$H(\mathbf{X })_{in} \ne H(\mathbf{X })_{out}$$.

where $H(\mathbf{X })_{in}$ and $H(\mathbf{X })_{out}$ denotes entropy distribution in in-degree and out-degree network respectively. We use $H_{0}$ and $H_{a}$ to denote the null hypothesis and alternate hypothesis following conventional notation. We perform a Kolmogorov–Smirnov (KS) test between the in-degree entropy distribution and out-degree entropy distribution to test our hypothesis. For all hypothesis tests, we use a significance level ($\alpha$) of 0.05. We find no statistically significant difference between the in-degree entropy distribution and out-degree entropy distribution with a KS statistic = 0.09 and *p* value = 0.81, thereby failing to reject the null hypothesis. Figure 5 illustrates entropy response and media likes distribution against varying media node-edge homogeneity for the in-degree network across all timestamps such that each timestamp is overlaid against the other. The comparison draws attention to the correlation between media distribution and entropy distribution in the in-degree network. To check for correlation, we compute the Pearson’s correlation coefficient between $\mathbf{M }^{t}$ and $\mathbf{H }^{t}$, where $\mathbf{H }^{t}$ represents network entropy distribution at time *t* while $\mathbf{M }^{t}$ represents media node-edge homogeneity of the network at time *t*. We compute the correlation for all $t \in T$ and for all users in the in-degree and out-degree network. The average correlation across all timestamps is $\rho = 0.82$, suggesting that entropy and media likes distribution are strongly positively correlated. Hence we hypothesize that the entropy in either network is sampled from the same distribution as the fake and authentic media distribution. Formally, we state our hypotheses as follows:

- $$H_{01}$$: $$H(\mathbf{X }) = T(\mathbf{Z })$$ and $$H_{02}$$: $$H(\mathbf{X }) = F(\mathbf{Z })$$
- $$H_{a1}$$: $$H(\mathbf{X }) \ne T(\mathbf{Z })$$ and $$H_{a2}$$: $$H(\mathbf{X }) \ne F(\mathbf{Z })$$

where $T(\mathbf{Z })$ and $F(\mathbf{Z })$ denotes the authentic and fake media distribution of the random variable $(\mathbf{Z })$ respectively. We find no statistically significant difference between entropy distribution and authentic media distribution ($H_{01}$) with a KS Statistic = 0.14 and *p* value = 0.28, thereby failing to reject the null hypothesis $H_{01}$. We also find no statistically significant difference between entropy distribution and fake media distribution ($H_{02}$) with a KS statistic = 0.12, *p* value = 0.46, thereby failing to reject the null hypothesis $H_{02}$. Thus, we observe that entropy response of media node-edge homogeneity and media likes distribution are strongly positively correlated. Though entropy response is individually correlated with authentic and fake media distribution, we hypothesize that it characterizes the superposition of both the media distributions. We assume and also empirically show (see Fig. 6a–c) that the underlying media likes distribution, as well as entropy distribution, are sampled from a Gaussian distribution such that media likes distribution is approximated as a linear combination of Gaussians, one describing the spread of fake media likes and the other describing the spread of authentic media likes as shown in Fig. 6d.

Based on our hypothesis, we assume that $\mathbf{H } \sim {\mathscr {N}}(\mu ,\,\sigma ^{2})$ is decomposable as a mixture of Gaussians such that $\mathbf{H }_{authentic} + \mathbf{H }_{fake} \sim {\mathscr {N}}(\mu _{authentic} + \mu _{fake},\,\sigma _{authentic}^{2} + \sigma _{fake}^{2})$, where each Gaussian component represents the underlying authentic and fake media likes distribution. Then we compute the Jensen–Shannon divergence (JSD)^59^ between the actual joint entropy distribution ($\mathbf{H }$) and Gaussian approximated entropy distributions ($\mathbf{H }_A$ and $\mathbf{H }_{B}$) as follows,

9 $$\begin{aligned} \begin{aligned} I(A)&= JSD(\mathbf{H }||\mathbf{H }_{A}\tilde{}{\mathscr {N}}(\mu _{A},\sigma _{A}^{2})) \\ I(B)&=JSD(\mathbf{H }||\mathbf{H }_{B}\tilde{}{\mathscr {N}}(\mu _{B},\sigma _{B}^{2})) \end{aligned} \end{aligned}$$

Hence, we formulate another pair of hypotheses:

- $$H_{01}:I(H ; {\mathscr {N}}(\mu _{authentic},\sigma _{authentic}^{2})) = I(H ; {\mathscr {N}}(\mu _{fake},\sigma _{fake}^{2}))$$
- $$H_{02}:I(A) = I(B)$$.

- $$H_{a1}:I(H ; {\mathscr {N}}(\mu _{authentic},\sigma _{authentic}^{2})) \ne I(H ; {\mathscr {N}}(\mu _{fake},\sigma _{fake}^{2}))$$
- $$H_{a2}:I(A) \ne I(B)$$.

To test our hypotheses, we use the Expectation-Maximization algorithm to estimate the Gaussian parameters and fit the data to the Gaussian components found using Gaussian mixture modeling. We perform a KS test between the information gain from authentic and fake media distribution as well as from the approximated Gaussian components. We reject the null hypothesis ($H_{01}$) that authentic media likes distribution is statistically significantly different from that of the fake media likes distribution with KS statistic = 1.0, *p* value = 9.99e$-16$ and the null hypothesis ($H_{02}$) that approximated authentic media likes distribution is statistically significantly different from that of the approximated fake media distribution with KS statistic = 1.0, *p* value = 8.61e$-83$ for the in-degree network.

Now, we describe how our model uses the results from the hypotheses tests to select user opinions as features for the media classifier. Once we compute the joint entropy response of the network to media node-edge homogeneity for varying $\gamma$, we approximate joint entropy as a mixture of Gaussians. We then compute the information gain from actual entropy and either of the Gaussian approximated media distributions and select the distribution with the maximum information gain (${\mathscr {Q}}$). Then, we define threshold ($\eta$) as a percentage of maximum user entropy in the network such that ${\mathscr {Q}}$ is the set of all users above $\eta$. Since the maximum entropy generated by a user is 4.0 bits, >25% refers to all users who have generated greater than 1.0 bit of entropy. We then compute an aggregated histogram of media likes per category from all users above the threshold. This enables us to aggregate all the media opinions of users in ${\mathscr {Q}}$ who have been selected as features for the media classifier. Finally, we compute the average media likes per category and predict media categories with media likes above that of the average media likes per category as authentic media, else fake. Algorithm box 2 depicts the media classification process in a step-by-step manner.

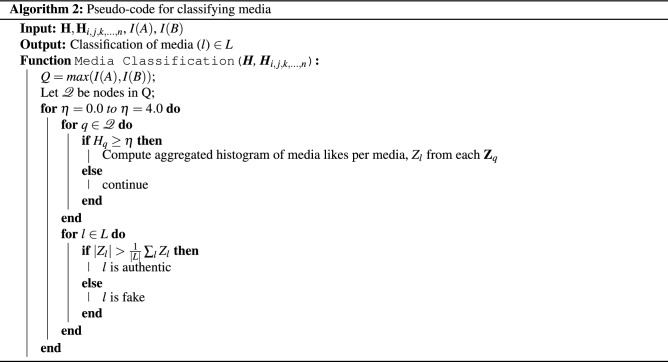



**Table 1:** Comparison of evaluation metrics.

Method	$\eta$	Precision	Recall	Accuracy	F1 score
Social network users	–	–	–	0.50	–
CNN & k-means (k=2)^60^	–	0.67	0.69	0.67	0.68
Ours	($>0$)%	0.83	0.50	0.50	0.63
Ours	($>25$)%	0.86	0.60	0.58	0.71
Ours	($>50$)%	0.88	0.70	0.67	0.78
Ours	($>75$)%	1.00	0.60	0.67	0.75

Since it has been shown that there exists a positive correlation between users and credibility, we test our model on the users from only the in-degree network. Apart from selecting users based on our model, we validate our work by benchmarking against (1) all users from the network as a classifier and (2) Durall et al. method^60^ which is a state-of-the-art unsupervised media classifier. From Table 1, we observe that using the combined human decision is similar to a random coin flip. Since we assume that media likes refers to user authentication^14^, other evaluation metrics such as F1 score, precision and recall, cannot be calculated from just media likes. In replicating Durall’s method, we compute feature embeddings for all media used in this experiment and consider this the test set. We use the convolutional neural network (CNN) architecture proposed by Durall et al. to generate media embeddings for the test set, which we use as input to the k-means clustering method with $k=2$. We find the F1 score of the unsupervised classifier to be outperformed by our model, as shown in Table 1. We find that selectively filtering users on the basis of a network independent measure such as entropy is sufficient to classify fake media with an accuracy higher than random chance and unsupervised state-of-the-art model. In a network where users are initially unknown to one another, we find that it is possible to approximate the underlying objective credibility using user opinions. Further, by computing the entropy response of the in-degree users using media node-edge homogeneity, we compute the entropy within the network. We approximate entropy as a superposition of fake and authentic media likes distribution. Since the distribution of fake media is different from authentic media, we are able to use the Gaussian approximated cluster with the highest information gain to utilize user opinions as features to classify fake media.

**Figure 7:**
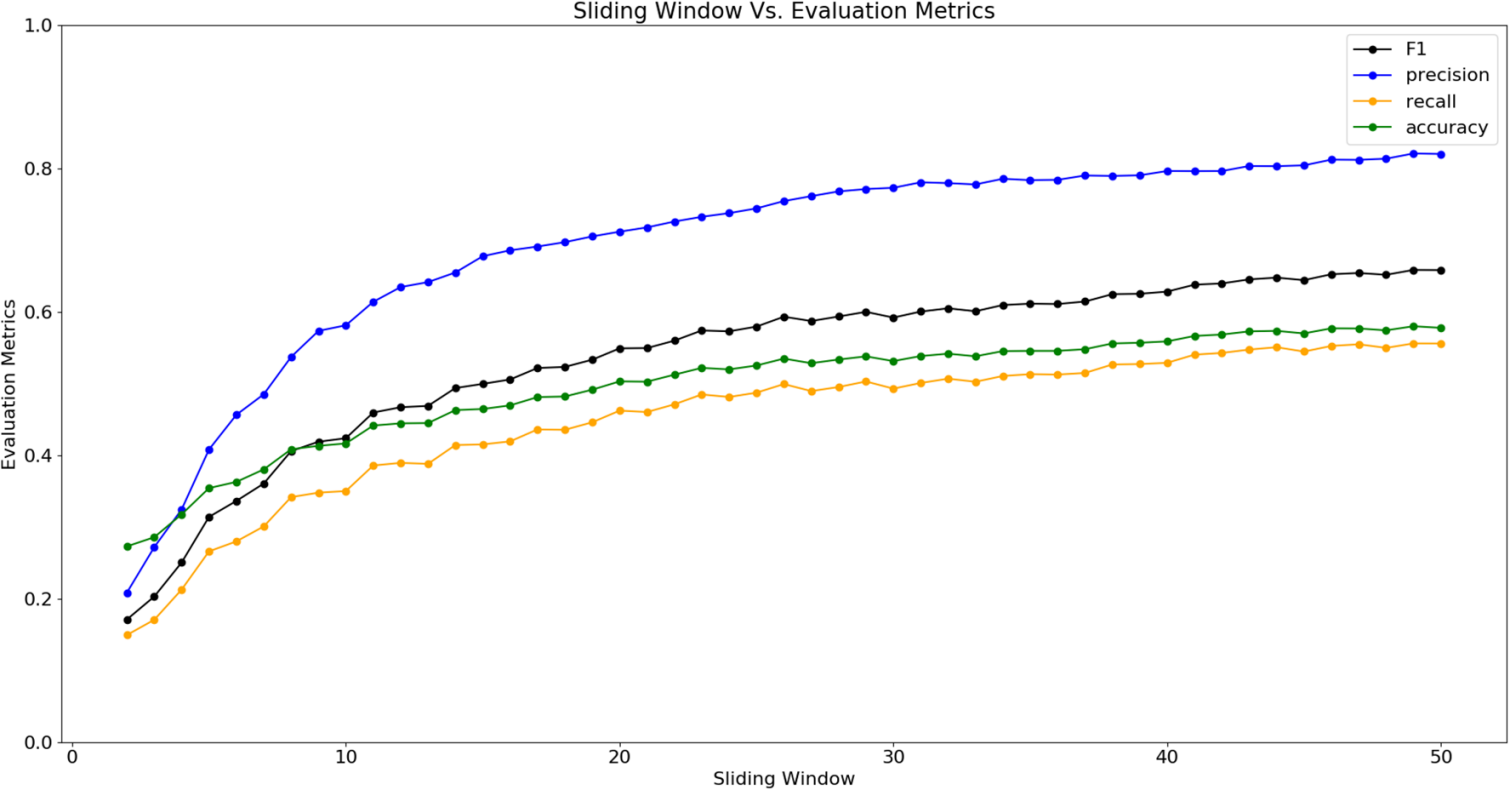
Sliding window versus evaluation accuracy.

### Time invariance of the proposed model

To study if our model is time-invariant to information classification, we perform an empirical experiment by introducing a sliding window parameter *h*. In Fig. 7, we illustrate the average performance of the classifier across all timestamps for every sliding window parameter in the range [2,50] and a stride parameter $s = 3$ which corresponds to the movement of the time window across time. We find that varying the time window varies the F1 score of the classifier with increasing time windows leading to increased F1, precision, recall and accuracy scores. When the sliding window parameter is increased by a value of 1 from 3 to 50, we approach the highest media classification accuracy and F1 score as shown in Table. 1. Since a time window of 2 corresponds to a period of 40 min in our study, a period of 17 h is required to achieve an F1 score of 0.68 which is comparable to the performance of the state-of-the-art unsupervised media classifier. Since social media networks have been operational for hundreds of thousands of hours, accessing prior time should not be an issue. In comparison, the state-of-the-art unsupervised approach proposed by Durall et al. requires neural network fine-tuning as new media is uploaded to the network or in the worst case, re-training, given the development of new media doctoring tools. Hence, while our model is not time-invariant, we show that it is possible for user–user and user–media interactions of a network over a period of time to be able to quantify the entropy needed to filter fake media.

Further, if we consider the relationship between time and entropy, we find that user activity in different timestamps often differs as a result of varying interaction with media (some network interactions happen at various timestamps with huge period of inactivity), causing entropy distribution of individuals to fluctuate with high uncertainty. However, when we consider 50 timestamps as shown in Fig. 7, the uncertainty due to user entropy fluctuations across different timestamps averages out, yielding consistent metric scores. This further highlights the dependence between time and the evolution of entropy.

## Conclusions

In this study, we design and conduct a controlled social media experiment with unknown human subjects in a network similar to Twitter. To characterize fake media using a network independent metric, we propose using media polarity and node-edge homogeneity, which are well-known detriments affecting the spread of fake media as a metric to compute the similarity between users in the network. In social media networks, interactions such as liking and sharing media, and forming/removing connections with other users, lead to the fluctuation of entropy over time. The entropy response of the in-degree users is captured using media node-edge homogeneity to compute the entropy within the network. In this paper, we compute entropy as a function of media polarized node-edge homogeneity to characterize the spread of fake and authentic media. Using our findings, we describe a majority voting classifier to classify online media using selective user opinions. However, our model is limited by the size of the moving window with the moving window size equal to that of the whole study improving the F1 score of the classifier. This shows that new users who join the network at different times are valuable for media classification. As future work, selective user opinions can be filtered using approaches such as active learning and combined with unsupervised media classifiers to further improve fake media classification in social media networks.

Apart from our findings, we highlight some of our assumptions that can possibly be grounded in theory using existing work or addressed as future work. We assume that no user knows each other prior to joining the network (random user network), media likes equal validation of authenticity, our 2-day study linearly maps to a longer-term study. Finally, future work may consider exploring the generalizability to other media types such as text and audio.

## Supplementary Information

Supplementary Information.

## Data Availability

The data along with the social media platform that was developed to collect the data as well as the corresponding algorithm have all been made available at the following repository: https://github.com/AiPEX-Lab/Social-Network-Analysis.

## References

[1] Li M, Wang X, Gao K, Zhang S (2017). A survey on information diffusion in online social networks: models and methods. Information.

[2] Marra, F., Gragnaniello, D., Cozzolino, D. & Verdoliva, L. Detection of GAN-generated fake images over social networks. In *2018 IEEE Conference on Multimedia Information Processing and Retrieval (MIPR)* (IEEE, 2018).

[3] Lazer DMJ (2018). The science of fake news. Science.

[4] Bergström A, Jervelycke Belfrage M (2018). News in social media: incidental consumption and the role of opinion leaders. Digit. Journal..

[5] Chesney B, Citron D (2019). Deep fakes: A looming challenge for privacy, democracy, and national security. Calif. L. Rev..

[6] Li L (2020). Characterizing the propagation of situational information in social media during covid-19 epidemic: A case study on weibo. IEEE Trans. Comput. Soc. Syst..

[7] Vaidyanathan G (2020). News Feature: Finding a vaccine for misinformation. Proc. Natl. Acad. Sci..

[8] Shu K, Sliva A, Wang S, Tang J, Liu H (2017). Fake news detection on social media: a data mining perspective. ACM SIGKDD Explor. Newslett..

[9] Fouss F, Francoisse K, Yen L, Pirotte A, Saerens M (2012). An experimental investigation of kernels on graphs for collaborative recommendation and semisupervised classification. Neural Netw..

[10] Parks L (2019). Dirty data: content moderation, regulatory outsourcing, and the cleaners. Film Q..

[11] Etlinger, S. What’s so difficult about social media platform governance?. *Models Platf. Gov.* **20**, (2019).

[12] Alhindi, T., Petridis, S. & Muresan, S. Where is your evidence: improving fact-checking by justification modeling. In *Proceedings of the first workshop on fact extraction and verification (FEVER)* **85–90**, (2018).

[13] Yang D (2018). True and fake information spreading over the Facebook. Phys. A Stat. Mech. Its Appl..

[14] Yang S (2019). Unsupervised fake news detection on social media: a generative approach. Proc. AAAI Conf. Artif. Intell..

[15] De Domenico M, Lima A, Mougel P, Musolesi M (2013). The anatomy of a scientific rumor. Sci. Rep..

[16] Filia A (2017). Ongoing outbreak with well over 4,000 measles cases in Italy from January to end August 2017- what is making elimination so difficult?. Eurosurveillance.

[17] Datta SS (2018). Progress and challenges in measles and rubella elimination in the WHO European Region. Vaccine.

[18] Tambuscio, M., Ruffo, G., Flammini, A. & Menczer, F. Fact-checking effect on viral hoaxes: a model of misinformation spread in social networks. In *Proceedings of the 24th International Conference on World Wide Web*, 977–982 (2015).

[19] Gupta, M., Zhao, P. & Han, J. Evaluating event credibility on twitter. In *Proceedings of the 2012 SIAM International Conference on Data Mining*, 153–164 (SIAM, 2012).

[20] Johnson TJ, Kaye BK (2015). Reasons to believe: influence of credibility on motivations for using social networks. Comput. Hum. Behav..

[21] Friggeri, A., Adamic, L. A., Eckles, D. & Cheng, J. Rumor cascades. In *ICWSM* (2014).

[22] Del Vicario M (2016). The spreading of misinformation online. Proc. Natl. Acad. Sci..

[23] Bovet A, Makse HA (2019). Influence of fake news in Twitter during the 2016 US presidential election. Nat. Commun..

[24] Vosoughi S, Roy D, Aral S (2018). The spread of true and false news online. Science.

[25] Stefanone, M. A., Vollmer, M. & Covert, J. M. In news we trust? Examining credibility and sharing behaviors of fake news. In *Proceedings of the 10th International Conference on Social Media and Society*, 136–147 (2019).

[26] Klayman J, Ha Y-W (1987). Confirmation, disconfirmation, and information in hypothesis testing. Psychol. Rev..

[27] Lou C, Yuan S (2019). Influencer marketing: how message value and credibility affect consumer trust of branded content on social media. J. Interact. Advert..

[28] Bandura A (2001). Social cognitive theory: an agentic perspective. Annu. Rev. Psychol..

[29] Golbeck, J. & Hendler, J. Filmtrust: movie recommendations using trust in web-based social networks. In *Proceedings of the IEEE Consumer Communications and Networking Conference*, vol. 96, 282–286 (Citeseer, 2006).

[30] Briscoe, E. J., Appling, D. S., Mappus IV, R. L. & Hayes, H. Determining credibility from social network structure. In *Proceedings of the 2013 IEEE/ACM International Conference on Advances in Social Networks Analysis and Mining*, 1418–1424 (2013).

[31] Scheufele DA, Krause NM (2019). Science audiences, misinformation, and fake news. Proc. Natl. Acad. Sci..

[32] Lim S, Tucker CS (2019). Mining Twitter data for causal links between tweets and real-world outcomes. Expert Syst. Appl. X.

[33] Wang, W. Y. “Liar, Liar Pants on Fire”: a new benchmark dataset for fake news detection. In *Proceedings of the 55th Annual Meeting of the Association for Computational Linguistics (Volume 2: Short Papers)*, 422–426 (2017).

[34] Shu, K., Wang, S. & Liu, H. Beyond news contents: the role of social context for fake news detection. In *Proceedings of the Twelfth ACM International Conference on Web Search and Data Mining* 312–320, (2019).

[35] Chu Z, Gianvecchio S, Wang H, Jajodia S (2012). Detecting automation of twitter accounts: are you a human, bot, or cyborg?. IEEE Trans. Dependable Secur. Comput..

[36] Sheng VS, Zhang J (2019). Machine learning with crowdsourcing: a brief summary of the past research and future directions. Proc. AAAI Conf. Artif. Intell..

[37] Long, C., Hua, G. & Kapoor, A. Active visual recognition with expertise estimation in crowdsourcing. In *Proceedings of the IEEE International Conference on Computer Vision*, 3000–3007 (2013). 10.1007/s11263-015-0834-9 PMC4764303 26924892

[38] Rodrigues, F., Pereira, F. & Ribeiro, B. Gaussian process classification and active learning with multiple annotators. In *International Conference on Machine Learning*, 433–441 (2014).

[39] Atarashi, K., Oyama, S. & Kurihara, M. Semi-supervised learning from crowds using deep generative models. In *Proceedings of the AAAI Conference on Artificial Intelligence*, vol. 32 (2018).

[40] Rodrigues, F. & Pereira, F. Deep learning from crowds. In *Proceedings of the AAAI Conference on Artificial Intelligence*, 32 (2018).

[41] Olmstead K, Mitchell A, Rosenstiel T (2011). Navigating news online: where people go, how they get there and what lures them away. Pew Res. Cent. Proj. Excell. Journal..

[42] An, J., Cha, M., Gummadi, K. & Crowcroft, J. Media landscape in Twitter: a world of new conventions and political diversity. In *Proceedings of the International AAAI Conference on Web and Social Media*, vol. 5 (2011).

[43] Hermida A, Fletcher F, Korell D, Logan D (2012). Share, like, recommend: decoding the social media news consumer. Journal. Stud..

[44] Van den Berg P, Wenseleers T (2018). Uncertainty about social interactions leads to the evolution of social heuristics. Nat. Commun..

[45] Zhao K, Karsai M, Bianconi G (2011). Entropy of dynamical social networks. PloS One.

[46] Zhao, Z., Resnick, P. & Mei, Q. Enquiring minds: early detection of rumors in social media from enquiry posts. In *Proceedings of the 24th International Conference on world wide web*, 1395–1405 (2015).

[47] Sinda, M. & Liao, Q. Spatial-temporal anomaly detection using security visual analytics via entropy graph and eigen matrix. In *2017 IEEE 15th International Conference on Dependable, Autonomic and Secure Computing, 15th International Conference on Pervasive Intelligence and Computing, 3rd International Conference on Big Data Intelligence and Computing and Cyber Science and Technology Congress (DASC/PiCom/DataCom/CyberSciTech*, 511–518. (IEEE, 2017). 10.1109/DASC-PICom-DataCom-CyberSciTec.2017.201 PMC6157906 30272054

[48] Shukla AS, Maurya R (2018). Entropy-based anomaly detection in a network. Wirel. Pers. Commun..

[49] Yang C (2019). Anomaly network traffic detection algorithm based on information entropy measurement under the cloud computing environment. Clust. Comput..

[50] Ahmed, S. & Tepe, K. Entropy-based recommendation trust model for machine to machine communications. In *Ad Hoc Networks*, 297–305. (Springer, 2017).

[51] Paryani, J., Ashwin Kumar, T. K., & George, K. M. Entropy-based model for estimating veracity of topics from tweets. In *International Conference on Computational Collective Intelligence*, 417–427. (Springer, 2017).

[52] Golbeck, J. *et al.* Fake news vs satire: a dataset and analysis. In *Proceedings of the 10th ACM Conference on Web Science*, 17–21 (2018).

[53] Rossler, A. *et al.* Faceforensics++: learning to detect manipulated facial images. In *Proceedings of the IEEE International Conference on Computer Vision*, 1–11 (2019).

[54] Rand DG, Arbesman S, Christakis NA (2011). Dynamic social networks promote cooperation in experiments with humans. Proc. Natl. Acad. Sci..

[55] Pennycook G, Rand DG (2019). Fighting misinformation on social media using crowdsourced judgments of news source quality. Proc. Natl. Acad. Sci..

[56] Hansen D, Shneiderman B, Smith MA, Himelboim I (2019). Analyzing Social Media Networks with NodeXL: Insights from a Connected World.

[57] Resnick P, Kuwabara K, Zeckhauser R, Friedman E (2000). Reputation systems. Commun. ACM.

[58] Josang, A. & Ismail, R. The beta reputation system. In *Proceedings of the 15th Bled Electronic Commerce Conference*, vol. 5, 2502–2511 (2002).

[59] Nielsen, F. A family of statistical symmetric divergences based on Jensen’s inequality. *arXiv preprint* arXiv:1009.4004 (2010).

[60] Durall, R., Keuper, M. & Keuper, J. Watch your up-convolution: CNN based generative deep neural networks are failing to reproduce spectral distributions. In *Proceedings of the IEEE/CVF Conference on Computer Vision and Pattern Recognition* 7890–7899, (2020).

