# Supplementary material for: Classification of unlabeled online media
Source: Sci Rep. 2021 Mar 25;11:6908. doi: 10.1038/s41598-021-85608-5 (PMC7994853; doi:10.1038/s41598-021-85608-5)
Supplement: Supplementary file 1 — Supplementary Information. [file 41598_2021_85608_MOESM1_ESM.pdf]

Classification of unlabeled online media

Sakthi Kumar Arul Prakash, Conrad Tucker

November 10, 2020

1 Data Collection

We create an experimental platform that replicates the functionality of Twitter social media network to collect data for our study. The network has an in-degree network and an out-degree network (followee network and follower network). The social media network was created using the open-sourced Django framework in Python and hosted on our project server. The purpose of the social media network is to simulate the inception and evolution of a network with initially unknown users to study the spread of fake information online. By doing so, we look to develop an unsupervised graphical model to classify fake media using selective user opinions. We recruit human subjects using Amazon MTurk, a platform for completing virtual tasks such as being a user in a social media network that requires human intelligence. To ensure that the human subjects are reliable in staying throughout the study and with a provision for new users to join, we only recruit subjects who have a HIT' (Human Intelligence Task) approval rate of greater than 90 % in all requesters' HITs. The users are further given a monetary incentive to stay in the experiment. We run the study until we do not observe any change such as video liking, sharing, commenting nor new links in the network. The study spanned a duration of two days with the entire user activity saved every 20 minutes, thus being the resolution of our time interval.

During the experiment, the recruited users are shown 40 videos, with 20 being fake videos and the remaining being authentic videos. Additionally, the users are allowed to form links with other users. To simulate the real-world scenario in Twitter where users follow users or are followed by users, thereby forming explicit connections Hansen et al. (2019), we introduce the notion of user credibility. According to Resnick et al. Resnick et al. (2000), reputation mechanisms provide an incentive for honest behavior and help users make decisions involving trust. In this study, we develop an approach to compute user credibility using trust modeling. Specifically, we use the Beta Reputation System (BRS) Josang and Ismail (2002) used

in e-commerce to compute users' credibility. We select this method as it is grounded in probability theory and uses expectations on probability distributions to derive the predicted benefit of trust to help users form explicit connections in a participatory media setting. Additionally, the chosen method is based on the beta probability density function, which can represent probability distributions of binary events.

2 Methods

2.1 User Credibility

In this study, we use the BRS model to compute user credibilities. The credibility of user i is C_i and we assume the prior probability of C_j^t to be generated from a Beta distribution with parameters a^t (prior authentic media category counts) and b^t (prior fake media category counts) such that $C_j^t \sim\ Beta(a^t,\ b^t)$. We use the uniform distribution as the prior to the Beta distribution such that every new user is assigned a credibility score of 0.1. As the user continues to form new links within the network, and like media, the credibility score gets updated. We use a Bayesian update rule to update the credibility score of the user at each timestamp. We state the credibility update rule for user i such that the parameter a^t is updated as $a_i^{t+1} \leftarrow a_i^t + a_i^{\delta t}$ and b^t is updated as $b_i^{t+1} \leftarrow b_i^t + b_i^{\delta t}$ such that,

$$C_i^{(t+1)} = \frac{a_i^t + a_i^{\delta t}}{a_i^t + a_i^{\delta t} + b_i^t + b_i^{\delta t}} \tag{1}$$

where δt is 20 minutes. To understand the relationship between credibility and follower count, we fit a regression model between follower counts and user credibility as shown in Fig. 1 and find the co-efficient of determination (R^2) as 0.56. This shows that there is a positive correlation between follower count and credibility score. Additionally, we also compute and illustrate the final credibility score distribution of the social media network at the last timestamp as shown in Fig. 2. We find that the distribution closely fits the Beta distribution given the uniform prior with $\mathbf{E}[C] = 0.1$.

2.2 Three dimensional illustration of media node-edge homogeneity, entropy and time

Fig. 3 and 4 illustrate the entropy response and media likes over time and media node-edge homogeneity respectively. Entropy and media likes attain steady-state over time.

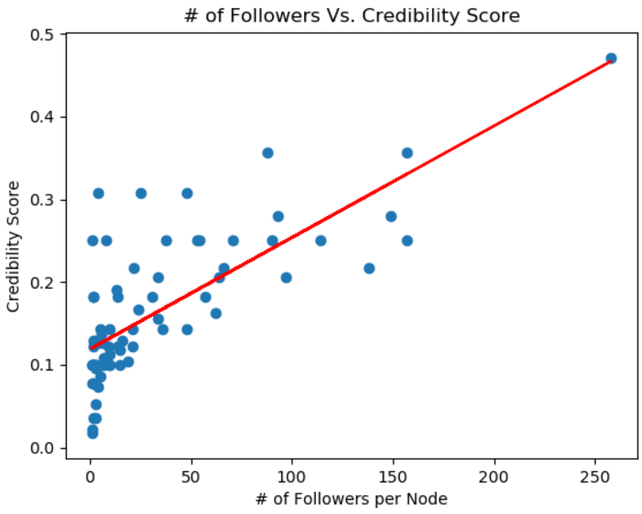

Figure 1: Fitted regression curve to follower counts against credibility score

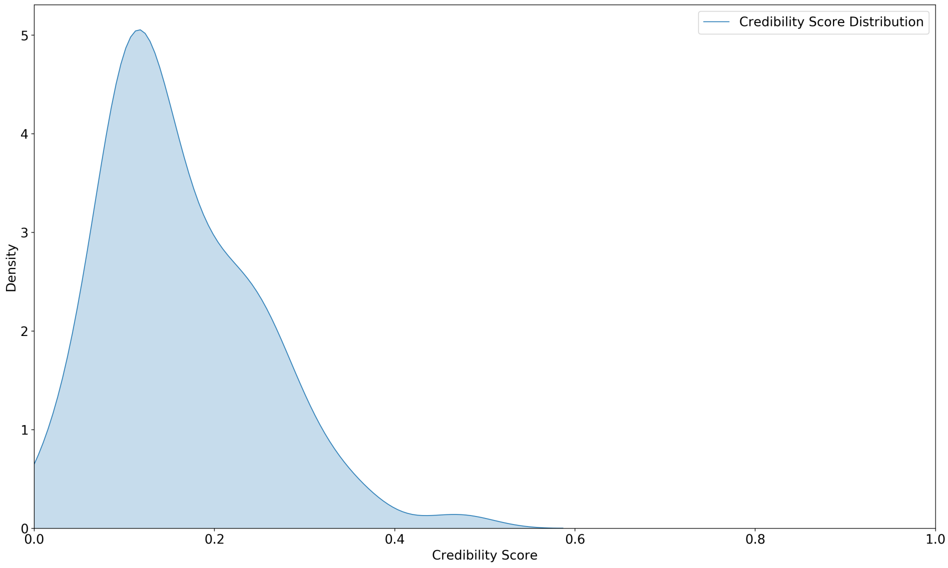

Figure 2: Credibility score distribution

3 Entropy approximation using Gaussian Mixture Model

Following entropy computation, we test a series of hypotheses to develop our model. In particular, we test the following hypotheses: Based on our hypothesis, we assume that $\mathbf{H} \sim \mathcal{N}(\mu, \sigma^2)$ is decomposable as a mixture of Gaussians such that $\mathbf{H}_{authentic} + \mathbf{H}_{fake} \sim \mathcal{N}(\mu_{authentic} + \mu_{fake}, \sigma^2_{authentic} +$

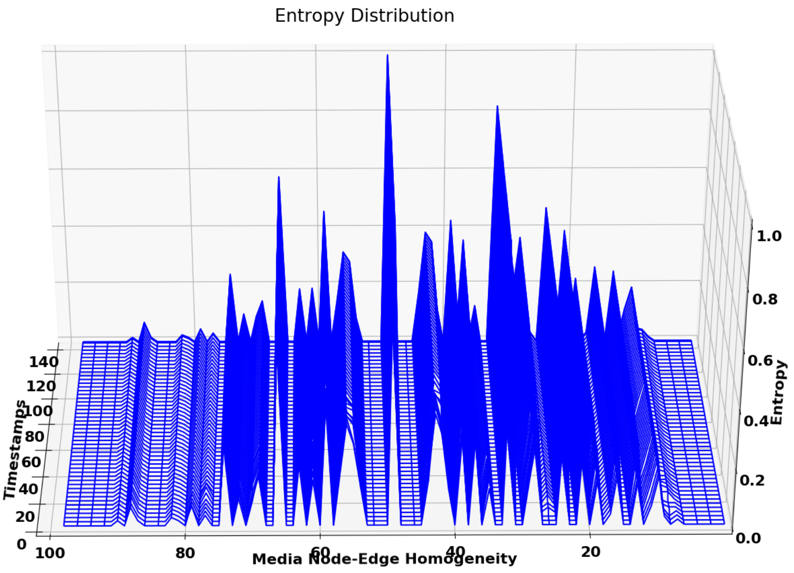

Figure 3: Entropy response of in-degree network in 3D

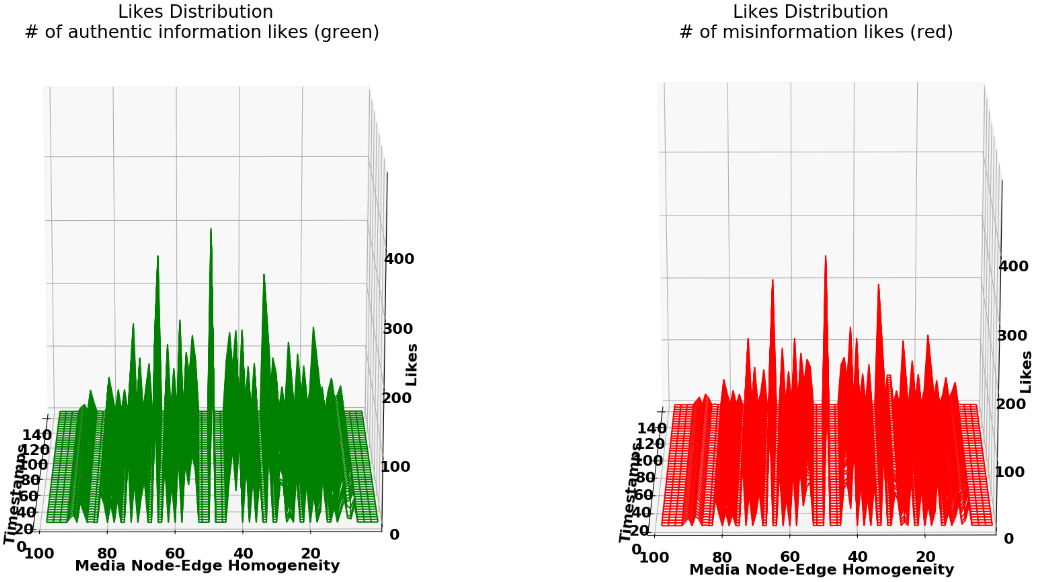

Figure 4: Media likes distribution of in-degree network in 3D

σ^2_{fake}) where each Gaussian component represents the underlying authentic and fake media likes distribution. Then we compute the Jensen–Shannon divergence (JSD) Nielsen, 2010 between the actual joint entropy distribu-

Table 1: Hypotheses

	Hypothesis	H_0	H_a
1	$H(\mathbf{X})_{in} = H(\mathbf{X})_{out}$	✓	
2	$H(\mathbf{X}) = T(\mathbf{Z})$ and $H(\mathbf{X}) = F(\mathbf{Z})$	✓	
3	$I(H; \mathcal{N}(\mu_{true}, \sigma^2_{true})) = I(H; \mathcal{N}(\mu_{False}, \sigma^2_{False}))$ and $I(H; \mathcal{N}(\mu_A, \sigma^2_A)) = I(H; \mathcal{N}(\mu_B, \sigma^2_B))$		✓

tion ($\mathbf{H}$) and Gaussian approximated entropy distributions ($\mathbf{H}_A$ and $\mathbf{H}_B$) as follows,

$$I(A) = JSD(\mathbf{H}||\mathbf{H}_A\tilde{\mathcal{N}}(\mu_A, \sigma_A^2)) \tag{2}$$

$$I(B) = JSD(\mathbf{H}||\mathbf{H}_B\tilde{\mathcal{N}}(\mu_B, \sigma_B^2)) \tag{3}$$

Since the information gain from authentic media is greater than fake media as seen in Fig. 3a and 3b from the results and discussion section in the main manuscript, we assume the distribution with a greater Jensen-Shannon divergence with actual entropy distribution to classify authentic information. We assign threshold intervals to utilize user opinions with increasing entropy as input features to a majority voting classifier to classify fake media.

References

Hansen, D et al. (2019). *Analyzing Social Media Networks with NodeXL: Insights from a Connected World*. Elsevier Science. ISBN: 9780128177570.

Josang, Audun and Roslan Ismail (2002). "The beta reputation system". In: *Proceedings of the 15th bled electronic commerce conference*. Vol. 5, pp. 2502–2511.

Nielsen, Frank (2010). "A family of statistical symmetric divergences based on Jensen's inequality". In: *arXiv preprint arXiv:1009.4004*.

Resnick, Paul et al. (2000). "Reputation systems". In: *Communications of the ACM* 43.12, pp. 45–48. ISSN: 0001-0782.
